# The Use of Medicines with Anti-cholinergic Properties and Their Health Impacts among Hospitalised Malaysian Geriatric Patients

**DOI:** 10.21315/mjms2019.26.2.9

**Published:** 2019-04-30

**Authors:** Izyan A Wahab, Bakht Akbar, Zainol Akbar Zainal, Mohd Farizh Che Pa, Basariah Naina

**Affiliations:** 1 Faculty of Pharmacy, Cyberjaya University College of Medical Sciences, Persiaran Bestari, Cyber 11, Cyberjaya, Selangor, Malaysia; 2 Pharmacy Department, Hospital Tuanku Ja’afar, Jalan Rasah, Bukit Rasah, Seremban, Negeri Sembilan, Malaysia

**Keywords:** anti-cholinergic, elderly, fall, confusion, length of stay

## Abstract

**Background:**

Studies have shown that the use of medicines with anti-cholinergic (Ach) properties can increase elderly patients’ risk of experiencing falls, confusion, and longer hospital stays (LOS). These adverse effects are preventable with appropriate intervention. Little is known about the use of medicines with Ach properties and their impact on Malaysian elderly patients. This study aimed to investigate the use of medicines with Ach properties and their impact on fall risk, confusion, and longer LOS among hospitalised elderly patients.

**Methods:**

This study utilised a cross-sectional design and was conducted at a single centre where convenience sampling was employed to collect data from elderly patients (> 60 years) admitted to geriatric and medical wards at Hospital Tuanku Ja’afar during a 2-month period (July 2017–August 2017). Patients were excluded from this study if their hospital admission was planned for an elective procedure or if neurocognitive and hepatic impairment were diagnosed prior to the hospital admission. Medicines with Ach properties were identified and classified according to the anti-cholinergic drug scale (ADS). Univariate and multiple logistic regression statistical analyses were performed to assess its impacts on falls, confusion, and LOS.

**Results:**

A total of 145 elderly patients with a mean age of 71.59 years old (SD = 8.02) were included in the study. Fifty-two percent of the participants were female, and the average hospital stay was 6 days (SD = 2.09). Medicines with Ach properties were administered in 62% (*n* = 90) of the cases. The most commonly prescribed medicine with Ach properties was furosemide (*n* = 59), followed by ranitidine (*n* = 44), warfarin (*n* = 23), and methylprednisolone (*n* = 22). Compared to patients who did not receive medicines with Ach properties, patients who received them had a significantly higher risk of falls [odds ratios (OR) = 2.61; 95%CI: 1.18, 5.78; *P* = 0.018], confusion (OR = 3.60; 95%CI: 1.55, 8.36; *P* = 0.003), and LOS (OR = 4.83; 95%CI: 2.13, 10.94; *P* < 0.001). Multiple comorbidities also showed a significantly increased risk of falls (OR = 3.03; 95%CI: 1.29, 7.07; *P* = 0.010).

**Conclusion:**

Medicines with Ach properties had a significant impact on elderly patients’ health. Strategies for rationally prescribing medicines with Ach properties to Malaysian elderly patients need to be improved and be recognised as an important public health priority.

## Introduction

Exposure to medicines with anti-cholinergic (Ach) properties is common among elderly (1, 2) patients. Approximately 50%–90% of elderly patients in Western countries are prescribed Ach medicines (3–5). The high rates of its use is a concern, especially for elderly patients who already have complex therapeutic regimens designed to manage multiple comorbidities. Many medicines with Ach properties that have been prescribed to the elderly have undesirable effects, including delirium, confusion, memory loss, and prolonged hospitalisation (6–9).

A wide range of medications have Ach effects, such as common medicines used to treat less-chronic disorders, for example, cough (diphenhydramine), runny nose (chlorpheniramine), and diarrhoea (diphenoxylate), and medicines used to treat chronic disorders, for example, heart failure (frusemide), atrial fibrillation (warfarin and digoxin), and depression (sertraline) (10). The cumulative exposure to multiple medicines with Ach properties is known as an Ach burden. The high burden of Ach medicines used to block acetylcholine’s action on its receptors, most of which are in the brain, has been shown to increase mortality and hospitalisation rates among elderly people (11).

Acetylcholine is an important neurotransmitter in the brain that regulates many activities in humans (12). The activation of these receptors is important for higher cognitive processes, such as the ability to comprehend a situation and make decisions. In the normal aging process, acetylcholine concentrations tend to decrease, and structural changes occur at the acetylcholine binding sites (12, 13). These changes may cause the elderly to experience sporadic short-term memory lapses and, hence, may have increased sensitivity to the blockade of cholinergic binding sites when exposed to Ach medicines.

No prior studies have been conducted in Malaysia to investigate the magnitude of prescribing medicines with Ach properties and their association with health outcomes, such as falls, confusion, and LOS. This study’s aim was to investigate the burden of medicines with Ach properties and their impact on patient health with respect to falls, confusion, and LOS.

## Methods

### The Study Design and Site

This study was a single centre, crosssectional retrospective study of elderly patients (age 60 or older) admitted between July 2017 and August 2017 (2 months) to the geriatric and medical wards of Hospital Tuanku Ja’afar, Seremban, which is one of Malaysia’s largest publicly funded hospitals with a total of 35 wards, 1070 beds, and 20 specialist clinics. This study obtained approval from the Malaysia Research and Ethics Committee (NMRR-17-1424-36317 [IIR]).

### Patient Selection Criteria and the Sample Size

Patients aged 60 or older were randomly selected if they had a complete medical record that included a demographic profile, the reason for hospitalisation, and the prescribed medications. Patients were excluded from this study if they had been diagnosed with a cognitive-psychiatry disease, stroke, or hepatic encephalopathy prior to the hospital admission. Patients admitted for elective surgery and investigational procedures were also excluded. The minimum sample size was calculated using an established sample size formula (14) from previous studies where *P* = 0.5 (3, 5). A minimum of 132 patients was required for this study. Hospital Tuanku Ja’afar has a bed occupancy rate (BOR) of 98% for medical wards. We used convenience sampling to select our elderly patients.

### Data Collection

Trained clinical researchers collected the following data from the selected patients’ medical records upon admission to the hospital’s geriatric and medical wards. Medicines with Ach properties were identified and classified according to the Ach load level using the anticholinergic drug scale (ADS) (15). This scale has been validated for medicines that have a significant association with serum Ach activity (15) and predicted Ach drug-related adverse events (16). The scales are as follows: Level 0 = no known Ach activity; Level 1 = potentially Ach activity as evidenced by receptor binding studies; Level 2 = Ach adverse events sometimes noted, usually at excessive doses; and Level 3 = markedly Ach. A list of medicines and their levels is included in Appendix 1. Adverse outcomes, such as falls and confusion (or delirium), as well as the LOS, were identified and extracted from patients’ medical records from admission until discharge. A previous history of these outcomes prior to admission was also identified and extracted. The LOS for all selected patients was counted from the first day of the hospital admission until discharge.

### Data Analysis

Descriptive statistics were used to summarise patients’ demographic variables. Continuous data were presented using means and standard deviations, and categorical data were presented as numbers and percentages. The prevalence of medicines with Ach properties was quantified over the two-month period, which was from 1 July 2017 to 31 August 2017. A univariate analysis was performed using a chi-squared test for categorical data. Variables used in the univariate analysis were age (0: 60–69 years, 1: ≥ 70 years), gender (0: man, 1: woman), smoking (1: Yes, 0: No), alcohol intake (1: Yes, 0: No), number of medicines prescribed (0: < 5 medicines, 1: ≥ 5 medicines), comorbidities

(1: Yes, 0: No), and Ach medicines prescribed (1: Yes, 0: No). Multiple logistic regressions were used and included the following variables; age, gender, comorbidities, number of medicines use, smoking, alcohol intake, and Ach medicine use to determine the predictors of adverse outcomes, which were fall, confusion, and longer LOS. All statistical analyses were undertaken using Stata v.12 (TX: StataCorp LP). The results are presented as odds ratios (OR) with 95% confidence intervals and *P*-values.

## Results

### Sample Characteristics

A total of 145 patients were included in this study. Of these, 52% (*n* = 75) were female, with a mean age of 71.59 years (SD = 8.02) (Table 1). Malay (54%, *n* = 79) was the most prevalent ethnicity in this study sample, followed by Chinese (28%, *n* = 28) and Indian (17%, *n* = 25). Most of the patients’ hospital admissions were due to symptoms, signs, and abnormal clinical and laboratory findings (72.4%, *n* = 105), followed by digestive system problems (12.4%, *n* = 18) (Table 2). Almost half of the patients had a history of falls (45%, *n* = 65); however, most of them had no history of fractures (95%, *n* = 137). For secondary diagnoses during the hospital stay, most of our sample had cardiovascular diseases (87%, *n* = 127). Of these, hypertension (32%, *n* = 41) and heart failure (30%, *n* = 30) were the most common diagnoses. The mean length of hospital stay was 6 days (SD = 2.09).

### The Utilisation of Medicines with Ach Properties

This study reveals that more than half of the patients took at least one medicine with Ach properties (62%, *n* = 90/145) during their hospitalisation, whereas 37% (*n* = 54/145) took a single medicine with Ach properties, and 25% (*n* = 36/145) were prescribed multiple medicines with Ach properties. Compared to non-users, individuals on Ach medicines were more likely to be aged > 70 years old and smoke tobacco, as well as have multiple comorbidities and more than five medicines in their regimen (Table 1). A higher number of medicines was associated with Ach medicine use. Twenty-one types of medicines with Ach properties were used by the geriatric patients (Table 3), for a total of 260 frequency exposures. Based on the ADS, the most common utilisation of medicines with Ach properties was Level 1-mild (81%, *n* = 209/260), followed by Level 2-moderate (17%, *n* = 44/260) and Level 3-severe (2.7%, *n* = 7/260).

Most of the medicines with Ach properties are cardiovascular system medications, such as furosemide (*n* = 59), warfarin (*n* = 23), digoxin (*n* = 13), and isosorbide dinitrate (*n* = 13). Respiratory medications were the second most commonly used medication with Ach properties. Among these respiratory medicines are prednisolone (*n* = 22), hydrocortisone (*n* = 16), theophylline (*n* = 11), and diphenhydramine (*n* = 6). All these medicines are ADS Level 1. Medication with Ach properties acting on the gastrointestinal system was the third most commonly used (i.e., ranitidine [*n* = 44]). This is the only ADS Level 2 medication used by the study patients. Hence, out of 145 patients, 44 (30%) were prescribed ranitidine. Only two ADS Level 3 medicines (i.e., diphenhydramine and chlorpheniramine) were used, by a total of 7 geriatric patients.

### Predictors of Falls, Confusion, and Length of Hospital Stay (LOS)

This study used correlation coefficient analysis to check for a multicollinearity threat. The correlation coefficient results showed the highest correlation coefficient is 0.3762, which is less than the 0.9000 cut-off point. Hence, there is no multicollinearity issue present in the study. The Hosmer-Lemeshow test results (Table 4) for all three models indicate that the *P*-values were not significant and that the three models’ fit is good.

The multiple regression analysis identified several patient factors associated with a high risk of falls, confusion, and LOS (Table 4). Medicines with Ach properties were significantly associated with a high risk of falls (OR = 2.61; 95%CI: 1.18, 5.78; *P* = 0.018), confusion (OR = 3.60; 95%CI: 1.55, 8.36; *P* = 0.003), and LOS (OR = 4.83; 95%CI: 2.13, 10.94; *P* = < 0.001). Comorbidities were also significantly associated with falls (OR = 3.03; 95%CI: 1.30, 7.08; *P* = 0.01). Gender, age, smoking, alcohol intake, and the number of medicines prescribed were not associated with any of the adverse outcome measures.

## Discussion

This paper provides a ‘snapshot’ of the pattern of Ach medicine use among geriatric patients at one hospital in Malaysia. The prevalence of Ach medicine use was high in this study, which is consistent with previous studies (17–19). We found that Ach medicine use was associated with falls, confusion, and longer hospital stays. The results of our study are similar to other studies, which also found an increased risk of falls (8, 17), cognitive impairment (18–20), and longer hospital stays (9).

Most patients in the present study had cardiovascular diseases, which reflects the high use of frusemide, a Level 1 drug on the ADS list. A Level 1 drug is potentially Ach as evidenced by receptor binding studies (14). Frusemide was among the top 20 drugs prescribed in Malaysia, along with other antihypertensive medicines, such as beta-blockers (i.e., atenolol and metoprolol) (21). Caution should be exercised when monitoring elderly patients who are prescribed frusemide because a large elderly cohort study conducted in Denmark has shown that the common antihypertensive agent is associated with fall-related fractures (22). Frusemide is commonly prescribed for oedema associated with congestive heart failure. It is often prescribed in combination with beta-blockers, which have also been associated with an increased risk of falls among elderly women (23). However, beta-blockers do not have significant Ach properties. The cumulative effect of frusemide, which has Ach properties, and beta-blockers is unknown.

A study was undertaken in Malaysia to determine the occurrence of polypharmacy in schizophrenia patients who were taking risperidone (24). The study found that Ach medicines were used in more than one-third of schizophrenia patients. The study, however, did not investigate the impact of Ach medicine use on these patients. Thus, our study addresses an important gap in the literature by evaluating the association between Ach medication use and fall risk, confusion, and LOS among a sample of elderly patients. Our study excluded patients with psychiatry illnesses that are already considered an independent risk factor for falls in the elderly (25, 26). Hence, the results of this study may underestimate the true magnitude of the problem.

Most of the Ach medicines used in our patient sample were of Level 1 (mild, potentially cholinergic). These medicines are used continuously, rather than intermittently, to treat chronic illnesses (Table 2). Our results are consistent with a study done among community-dwelling elderly in Australia with and without dementia (27). The study found that the level-1 Ach medicines contributed to the highest Ach load where cardiovascular medicines were the most commonly prescribed including furosemide, warfarin and isosorbide mononitrate. Cumulative Ach medicines use have shown to cause adverse drug reactions (28) and increase hospitalisation and mortality in the elderly (9). Our study did not measure the cumulative effect of each level of Ach medicine on falls, confusion, and LOS. However, given the findings of our study, further consistency of results can be expected because a higher number of medicines used was associated with Ach medicine intake (Table 1). This result is also supported by another study where the number of medicines used was a predictor of a higher Ach burden (29). The relatively large number of Ach medicines categorised as Level 1 or low Ach potency needs to be made communicated to clinicians because the risk of falls, confusions, and longer LOS for one Level 3 Ach medicine may be the same as when multiple Level 1 Ach medicines are used.

Although only a handful of Levels 2 and 3 Ach medicines were prescribed in this study sample (i.e., ranitidine, diphenhydramine, and chlorpheniramine), caution should be exercised because these medicines can easily alter elderly patients’ mental status, especially in the emergency department (ED) where confusion is a common presentation and the chief complaint among elderly patients (30). A study involving 426 patients in the United States found that diphenhydramine administration during hospitalisation resulted in an increased risk of delirium symptoms, hallucinations, and altered consciousness (31).

Efforts to reduce the Ach medicines use in older people is currently underway. Reducing the dose or discontinuing Ach medicines are some of the awareness and implementation protocols currently being executed by clinical pharmacists (32–34). Other tools and measures are also being investigated to improve medication use among elderly patients in various settings (35–37). From the perspective of healthcare practitioners, drug developers, and regulators, establishing a common ground of understanding regarding pertinent information about older people for rational prescribing may still be lacking, and further collaboration among these stakeholders is highly recommended to improve prescribing in this population (38). Regardless of the various proposed strategies, no single method or approach could guarantee medication safety in older populations at any one time. Medication safety in older people will require simultaneous changes and optimisation in information handling, drug distribution and education, prescription, follow-up routines, and tools (39).

In this study, we did not make adjustments to account for potential confounders that could occur when there are differences between the Ach medicine exposed and non-exposed groups. This study shows that there are no significant differences between the two groups (Table 1), and the variables tested are not related to each other in the linear regression models. The results of this study, however, cannot be generalised to all geriatric patients in Malaysia because only a few outcomes were assessed within a short period of time, and we did not control for the dose and duration of Ach medicine use. Other factors that could contribute to falls, confusions and longer LOS, such as low bone mineral density, dehydration, and renal function status, were not taken into consideration in this study. Future work is required to include more factors and investigate Ach medicine doses that could be safely used in the elderly when no alternatives can be recommended.

## Conclusion

A significant percentage of our sample of elderly patients were exposed to Ach medicines. The use of medicines with Ach properties was associated with falls, confusion, and longer LOS. Level 1 Ach medicines were commonly used in our sample. Healthcare providers are highly encouraged to be more cautious in prescribing medicines to older, hospitalised patients. Since many over-the-counter and prescribed medicines have Ach properties, a continuous effort by all healthcare providers and authorities to educate older people and their caretakers should be established as an important public health priority.

## Figures and Tables

**Table 1:** Characteristics of sample: Overall and by Ach use (*n* = 145)

	Overall		User of Ach		Non-user of Ach		*X^2^-*statistics (df)^a^	*P*-value^a^
		
	(*n* = 145)		(*n* = 90)		(*n* = 55)			
		
	*N*	%	*N*	%	*N*	%		
Gender								
Male	70	48.3	45	50.0	25	45.5	0.282 (1)	0.595
Female	75	51.7	45	50.0	30	54.5		
Age (Mean (SD) = 71.51 (8.02)								
60–69	67	46.2	39	43.3	28	50.9	0.788 (1)	0.375
≥ 70	78	53.8	51	56.7	27	49.1		
Smoking								
No	126	86.9	79	87.7	47	85.5	0.162 (1)	0.687
Yes	19	13.1	11	12.3	8	14.5		
Alcohol intake								
No	134	92.4	84	93.3	50	91.0	0.286 (1)	0.593
Yes	11	7.6	6	6.7	5	9.0		
Comorbidities								
No	39	26.9	20	22.2	19	34.5	2.637 (1)	0.104
Yes	106	73.1	70	77.8	36	65.5		
Number of medicines								
< 5	68	46.9	29	32.2	39	70.9	20.516 (1)	<0.001^*^
≥ 5	77	53.1	61	67.8	16	29.1		

a Chi-square test for independence, *P* <0.05 for significance level

**Table 2:** Cause of hospital admission of elderly patients samples (*n* = 145)

Cause of Admission^*^	Frequency (%) *n* = 145
Symptoms, signs and abnormal clinical and laboratory findings, not elsewhere classified	105 (72.4)
Diseases of the digestive system	18 (12.4)
Certain infectious and parasitic diseases	9 (6.2)
External causes of morbidity and mortality	8 (5.5)
Diseases of the circulatory system	5 (3.5)

**Table 3:** Prescribed medicines with Ach properties

Medicine	ADS Level	Number of Patients
Furosemide	1	59
Ranitidine	2	44
Warfarin	1	23
Methylprednisolone	1	22
Hydrocortisone	1	16
Digoxin	1	13
Isosorbide dinitrite	1	13
Tramadol	1	13
Theophylline	1	11
Ampicillin	1	11
Vancomycin	1	7
Piperacillin	1	6
Diphenhydramine	3	6
Alprazolam	1	4
Lorazepam	1	4
Morphine	1	3
Diazepam	1	1
Dexamethasone	1	1
Captopril	1	1
Clindamycin	1	1
Cholorpheniramine	3	1

**Table 4:** Association of fall, confusion and LOS with tested variables

Variables	Fall		Confusion		LOS	
	
	OR (95%CI)	*P*-value	OR (95%CI)	*P*-value	OR (95%CI)	*P*-value
Age	1.06 (0.52–2.16)	0.864	1.44 (0.70–2.97)	0.320	0.98 (0.47–2.02)	0.952
Gender	0.70 (0.33–1.45)	0.335	0.74 (0.35–1.57)	0.427	0.56 (0.26–1.18)	0.128
Comorbidities	3.03 (1.30–7.08)*	0.010*	1.46 (0.63–3.38)	0.380	0.56 (0.25–1.29)	0.175
Number of medicines use	0.72 (0.34–1.56)	0.410	0.78 (0.36–1.69)	0.524	1.10 (0.51–2.38)	0.808
Smoking	0.63 (0.21–1.90)	0.410	1.55 (0.50–4.80)	0.444	0.75 (0.24–2.36)	0.622
Alcohol intake	1.21 (0.31–4.71)	0.788	0.32 (0.06–1.66)	0.176	0.47 (0.11–1.94)	0.295
Anticholinergic medicine use	2.61 (1.18–5.78)*	0.018*	3.60 (1.55–8.37)*	0.003*	4.83 (2.14–10.94)*	< 0.001*
Hosmer and Lemeshow (Prob > chi^2^)	0.521		0.213		0.754	

* Statistically significant

**Appendix 1:** Drugs classification based on ADS levels (available in Hospital Tuanku Ja’afar)

**Level 3 Drugs**		

Amitriptyline	Clozapine	Meclizine
Atropine	Diphenhydramine	Oxybutynin
Chlorpheniramine	hydroxyzine	Promethazine
Chlorpromazine	Imipramine	Tolterodine
Clomipramine		

**Level 2 Drugs**		

Carbamazepine	Ranitidine	

**Level 3 Drugs**		

Alprazolam	Fluoxetine	Nifedipine
Amantadine	Fluphenazine	Olanzapine
Ampicillin	Fluticasone-salmeterol	Oxycodone
Azathioprine	Fluvoxamine	Pancuronium
Bromocriptine	Furosemide	Phenelzine
Captopril	Gentamicin	Piperacillin
Clindamycin	Hydralazine	Prednisolone
Clonazepam	Hydrocortisone	Prochlorperazine
Cycloserine	Isosorbide	Sertralline
Cyclosporine	Isosorbide dinitrate	Theophylline
Dexamethasone	Isosorbide mononitrate	Tramadol
Diazepam	Loperamide	Triamcinolone
Digoxin	Lorazepam	Valproic acid
Diltiazem	Methylprednisolone	Vancomycin
Dipyridamole	Midazolam	Warfarin
Fentanyl	Morphine	
